# Crystal structure of 2-{(*R*)-[1-(4-bromo­phen­yl)eth­yl]imino­meth­yl}-4-(phenyl­diazen­yl)phenol, a chiral photochromic Schiff base

**DOI:** 10.1107/S2056989015019866

**Published:** 2015-10-28

**Authors:** Ryoji Moriwaki, Takashiro Akitsu

**Affiliations:** a Department of Chemistry, Faculty of Science, Tokyo University of Science, 1-3 Kagurazaka, Shinjuku-ku, Tokyo 162-8601, Japan

**Keywords:** crystal structure, Schiff base, azo­benzene, photochromic

## Abstract

The title chiral photochromic Schiff base compound, C_21_H_18_BrN_3_O, was synthesized from (*R*)-(+)-1-(4-bromo­phen­yl)ethyl­amine and the salicyl­aldehyde of an azo­benzene derivative. The mol­ecule corresponds to the phenol–imine tautomer, the C=N and N—C bond distances being 1.285 (3) and 1.470 (3) Å, respectively. The diazenyl group adopts a *trans* form, with an N=N distance of 1.256 (3) Å. The hy­droxy group is involved in intra­molecular O—H⋯N hydrogen bonding. In the crystal, C—H⋯π inter­actions consolidate the crystal packing of one-dimensional chains, which exhibits short inter­molecular Br⋯C contacts of 3.400 (3) Å.

## Related literature   

For applications of (chiral) photochromic Schiff base compounds, see: Akitsu & Einaga (2006*b* ▸); Akitsu *et al.* (2004 ▸); Aritake *et al.* (2010 ▸); Miura *et al.* (2009 ▸). For the crystal structures of related compounds, see: Akitsu & Einaga (2005*a* ▸,*b* ▸, 2006*a* ▸); Akitsu (2007 ▸); Akitsu & Itoh (2010 ▸); Aslantas *et al.* (2007 ▸); Hadjoudis & Mavridis (2004 ▸); Khandar & Rezvani (1999 ▸).
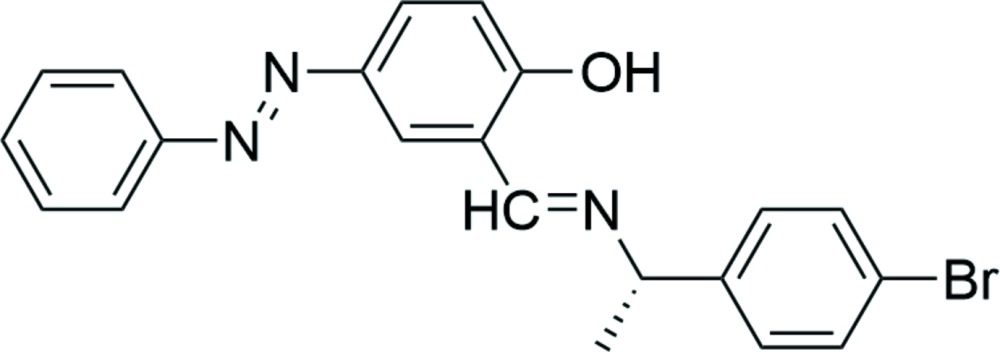


## Experimental   

### Crystal data  

C_21_H_18_BrN_3_O
*M* *_r_* = 408.29
Orthorhombic 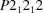

*a* = 7.271 (3) Å
*b* = 41.901 (15) Å
*c* = 5.952 (2) Å
*V* = 1813.3 (11) Å^3^
*Z* = 4
Mo *K*α radiation
μ = 2.28 mm^−1^
*T* = 113 K
0.37 × 0.23 × 0.08 mm

### Data collection  

Bruker APEXII CCD diffractometer
Absorption correction: empirical (using intensity measurements) (*SADABS*; Sheldrick, 1996 ▸) *T*_min_ = 0.486, *T*_max_ = 0.833
10349 measured reflections
4176 independent reflections
3723 reflections with *I* > 2σ(*I*)
*R*_int_ = 0.027

### Refinement  

*R*[*F* ^2^ > 2σ(*F* ^2^)] = 0.026
*wR*(*F* ^2^) = 0.047
*S* = 1.01
4176 reflections
237 parameters
H-atom parameters constrained
Δρ_max_ = 0.57 e Å^−3^
Δρ_min_ = −0.40 e Å^−3^
Absolute structure: Flack *x* determined using 1360 quotients [(*I* ^+^) − (*I* ^−^)]/[(*I* ^+^) + (*I* ^−^)] (Parsons *et al.*, 2013 ▸)
Absolute structure parameter: 0.005 (4)

### 

Data collection: *APEX2* (Bruker, 1998 ▸); cell refinement: *SAINT* (Bruker, 1998 ▸); data reduction: *SAINT*; program(s) used to solve structure: *SHELXS97* (Sheldrick, 2008 ▸); program(s) used to refine structure: *SHELXL2014* (Sheldrick, 2015 ▸); molecular graphics: *SHELXTL* (Sheldrick, 2008 ▸); software used to prepare material for publication: *SHELXTL*.

## Supplementary Material

Crystal structure: contains datablock(s) global, I. DOI: 10.1107/S2056989015019866/cv5499sup1.cif

Structure factors: contains datablock(s) I. DOI: 10.1107/S2056989015019866/cv5499Isup2.hkl

Supporting information file. DOI: 10.1107/S2056989015019866/cv5499Isup3.cml

**Fig. 1.**
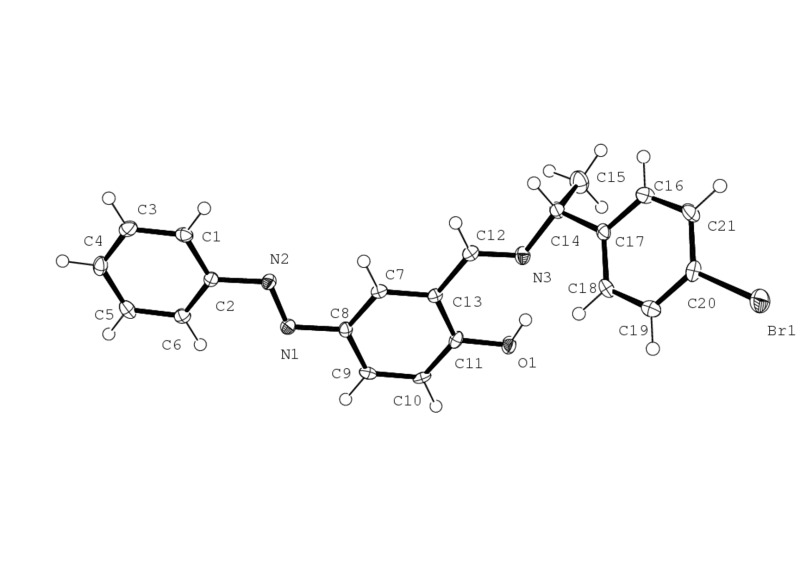
Molecular structure of (I) showing the atomic numbering and 50% probability displacement ellipsoids.

. DOI: 10.1107/S2056989015019866/cv5499fig1.tif
Mol­ecular structure of (I) showing the atomic numbering and 50% probability displacement ellipsoids.

CCDC reference: 1432412

Additional supporting information:  crystallographic information; 3D view; checkCIF report

## Figures and Tables

**Table 1:** Hydrogen-bond geometry (, ) *Cg*1 and *Cg*2 are centroids of C1C6 and C7C11/C13 rings, respectively.

*D*H*A*	*D*H	H*A*	*D* *A*	*D*H*A*
O1H1*A*N3	0.84	1.84	2.585(3)	148
C12H12*Cg*1^i^	0.95	2.80	3.399(3)	122
C10H10*Cg*1^ii^	0.95	2.74	3.415(3)	128
C6H6*Cg*2^iii^	0.95	2.75	3.423(3)	128

Symmetry codes: (i) 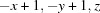; (ii) 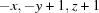; (iii) 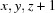.

## supplementary crystallographic information

### S1. Comment

In recent years, there is a growing interest in the organic/inorganic metal complexes and photochromic compounds. For example, *cis*-*trans* photoisomerization of azobenzene could switch conformation of chiral ligands (Akitsu & Einaga, 2005*a*, 2005*b*), chiral conformation change in a solution induced by a photochromic solute (Akitsu & Einaga, 2006*a*; Akitsu, 2007) and optical anisotropy in polymeric films (Akitsu & Itoh, 2010). Also free Schiff base ligands may act as photochromic, thermochromics, and fluorescence materials (Akitsu *et al.*, 2004; Hadjoudis & Mavridis, 2004; Akitsu & Einaga, 2006*b*). Recently, we have synthesized the title compound (I). Herewith we present its crystal structure.

The molecule of (I) (Fig. I) adopts an E configuration with respect to the imine C═N double bond with C13—C12—N3—C14 torsion angle of 178.6 (2) °. Thus, the π-conjugated system around the imine group is essentially planar. All bond lengths and angles in (I) correspond well to those observed in similar Schiff base ligands (Akitsu & Einaga, 2006*b*; Miura *et al.*, 2009; Aritake *et al.*, 2010) and azobenzene derivatives (Aslantas *et al.*, 2007; Khandar & Rezvani, 1999). The C11—O1 bond distance of 1.350 (3) Å suggests that it is the phenol-imine tautomer. The contraction of the C12═N3 bond [1.285 (3) Å] is also in agreement with the phenol-imine tautomer. As for the azobenzene moiety, the azo N═N double bond adopts an E configuration with the N═N distance of 1.256 (3) Å. Hydroxyl group is involved in intramolecular O—H···N hydrogen bond (Table 1).

In the crystal, C—H···π interactions (Table 1) consolidate the crystal packing, which exhibits short intermolecular Br1···C20(1/2+x, 1/2-y, 2-z) contact of 3.400 (3) Å.

### S2. Experimental

Treatment of aniline (0.951 g, 10.0 mmol) in 15 ml of 6*M* HCl and NaNO_2_ (0.690 g, 10 mmol) in 15 ml of H_2_O for 30 min at 278 K gave rise to the yellow precursor. Treatment of the precursor and salicylaldehyde (1.22 g 10 mmol) in 30 ml of 10% NaOH aqueous solution for 1 h at 278 K, and the resulting brown precipitates were filtrated and washed with water and ethanol, and dried in a desiccator for several days. Treatment of the brown precipitates (0.226 g, 1.00 mmol) and (*R*)-(+)-1-(4-Bromophenyl)ethylamine (0.200 g, 1.00 mmol) for 2 h at 298 K under a nitrogen atmosphere gave rise to orange compound after evaporation(yield 0.0598 g, 29.3%). This crude orange compound was filtered and recrystallized slow evaporation from aceton to give orange prismatic single crystals. Anal. Calc. for C_21_H_18_BrN_3_O: C, 61.78; H, 4.44; N, 10.29. Found: C, 61.66; H, 4.67; N, 10.17%. IR (KBr,(cm-1)): 1585 (N=N), 1632(C=N). ^1^H NMR (300 MHz, DMSO) δ(p.p.m.): 1.60 (d,3*H*), 2.50 (m,2*H*), 4.80 (dd,1*H*), 7.03 (d,2*H*), 7.41 (tt,2*H*), 7.56 (m,5*H*), 7.84 (tt,2*H*), 7.95 (dd,1*H*), 8.12 (d,1*H*), 8.88(s,1*H*).

### S3. Refinement

All hydrogen atoms were geometrically positioned and refined as riding.

### Crystal data

**Table:**

C_21_H_18_BrN_3_O	*D*_x_ = 1.496 Mg m^−^^3^
*M**_r_* = 408.29	Mo *K*α radiation, λ = 0.71073 Å
Orthorhombic, *P*2_1_2_1_2	Cell parameters from 10349 reflections
*a* = 7.271 (3) Å	θ = 2.8–27.7°
*b* = 41.901 (15) Å	µ = 2.28 mm^−^^1^
*c* = 5.952 (2) Å	*T* = 113 K
*V* = 1813.3 (11) Å^3^	Needle, orange
*Z* = 4	0.37 × 0.23 × 0.08 mm
*F*(000) = 832	

### Data collection

**Table:**

Bruker APEXII CCD diffractometer	4176 independent reflections
Radiation source: fine-focus sealed tube	3723 reflections with *I* > 2σ(*I*)
Detector resolution: 8.333 pixels mm^-1^	*R*_int_ = 0.027
φ and ω scans	θ_max_ = 27.7°, θ_min_ = 2.8°
Absorption correction: empirical (using intensity measurements) (*SADABS*; Sheldrick, 1996)	*h* = −8→9
*T*_min_ = 0.486, *T*_max_ = 0.833	*k* = −54→33
10349 measured reflections	*l* = −7→7

### Refinement

**Table:**

Refinement on *F*^2^	Secondary atom site location: difference Fourier map
Least-squares matrix: full	Hydrogen site location: inferred from neighbouring sites
*R*[*F*^2^ > 2σ(*F*^2^)] = 0.026	H-atom parameters constrained
*wR*(*F*^2^) = 0.047	*w* = 1/[σ^2^(*F*_o_^2^)] where *P* = (*F*_o_^2^ + 2*F*_c_^2^)/3
*S* = 1.01	(Δ/σ)_max_ = 0.002
4176 reflections	Δρ_max_ = 0.57 e Å^−^^3^
237 parameters	Δρ_min_ = −0.40 e Å^−^^3^
0 restraints	Absolute structure: Flack *x* determined using 1360 quotients [(I+)-(I-)]/[(I+)+(I-)] (Parsons *et al.*, 2013)
Primary atom site location: structure-invariant direct methods	Absolute structure parameter: 0.005 (4)

### Special details

**Table:**

Geometry. All e.s.d.'s (except the e.s.d. in the dihedral angle between two l.s. planes) are estimated using the full covariance matrix. The cell e.s.d.'s are taken into account individually in the estimation of e.s.d.'s in distances, angles and torsion angles; correlations between e.s.d.'s in cell parameters are only used when they are defined by crystal symmetry. An approximate (isotropic) treatment of cell e.s.d.'s is used for estimating e.s.d.'s involving l.s. planes.

### Fractional atomic coordinates and isotropic or equivalent isotropic displacement parameters (Å^2^)

**Table:**

	*x*	*y*	*z*	*U*_iso_*/*U*_eq_	
Br1	1.09221 (4)	0.26333 (2)	0.94955 (5)	0.02934 (9)	
C1	0.2882 (4)	0.55533 (6)	0.2777 (4)	0.0162 (6)	
H1	0.347	0.54	0.185	0.019*	
C2	0.2286 (3)	0.54700 (5)	0.4928 (4)	0.0132 (6)	
C3	0.2610 (4)	0.58613 (6)	0.2004 (5)	0.0191 (6)	
H3	0.3033	0.5919	0.0551	0.023*	
C4	0.1732 (4)	0.60841 (6)	0.3323 (5)	0.0193 (7)	
H4	0.1531	0.6294	0.2766	0.023*	
C5	0.1136 (4)	0.60022 (5)	0.5478 (5)	0.0189 (6)	
H5	0.0549	0.6157	0.6397	0.023*	
C6	0.1400 (3)	0.56948 (5)	0.6277 (4)	0.0153 (6)	
H6	0.098	0.5638	0.7733	0.018*	
C7	0.3225 (3)	0.45296 (5)	0.6999 (4)	0.0130 (6)	
H7	0.3679	0.4579	0.5543	0.016*	
C8	0.2539 (4)	0.47727 (5)	0.8343 (4)	0.0133 (6)	
C9	0.1948 (3)	0.47027 (5)	1.0517 (5)	0.0147 (5)	
H9	0.1538	0.487	1.1473	0.018*	
C10	0.1954 (4)	0.43923 (6)	1.1290 (4)	0.0147 (6)	
H10	0.1524	0.4347	1.2762	0.018*	
C11	0.2586 (3)	0.41462 (5)	0.9931 (4)	0.0152 (6)	
C12	0.3926 (4)	0.39625 (5)	0.6274 (4)	0.0142 (5)	
H12	0.4383	0.4015	0.4826	0.017*	
C13	0.3259 (4)	0.42154 (5)	0.7748 (4)	0.0120 (6)	
C14	0.4556 (3)	0.34250 (5)	0.5321 (5)	0.0170 (6)	
H14	0.5028	0.3533	0.394	0.02*	
C15	0.2920 (4)	0.32168 (6)	0.4678 (5)	0.0273 (7)	
H15A	0.2462	0.3106	0.6014	0.041*	
H15B	0.3305	0.306	0.3551	0.041*	
H15C	0.1941	0.3351	0.4054	0.041*	
C16	0.6884 (4)	0.29788 (5)	0.5224 (5)	0.0206 (6)	
H16	0.6402	0.2924	0.3791	0.025*	
C17	0.6118 (4)	0.32344 (5)	0.6378 (4)	0.0148 (6)	
C18	0.6859 (4)	0.33094 (6)	0.8466 (4)	0.0212 (7)	
H18	0.637	0.3484	0.9289	0.025*	
C19	0.8302 (4)	0.31341 (5)	0.9371 (5)	0.0220 (6)	
H19	0.8799	0.3189	1.0796	0.026*	
C20	0.9003 (4)	0.28803 (5)	0.8183 (4)	0.0186 (6)	
C21	0.8323 (4)	0.28005 (6)	0.6082 (5)	0.0214 (7)	
H21	0.8831	0.2628	0.5253	0.026*	
N1	0.2355 (3)	0.50990 (4)	0.7656 (4)	0.0149 (5)	
N2	0.2565 (3)	0.51440 (4)	0.5585 (4)	0.0150 (5)	
N3	0.3906 (3)	0.36692 (4)	0.6905 (3)	0.0169 (5)	
O1	0.2552 (3)	0.38445 (4)	1.0728 (3)	0.0200 (4)	
H1A	0.2988	0.372	0.9758	0.03*	

### Atomic displacement parameters (Å^2^)

**Table:**

	*U*^11^	*U*^22^	*U*^33^	*U*^12^	*U*^13^	*U*^23^
Br1	0.03065 (17)	0.02551 (13)	0.03187 (16)	0.01116 (13)	−0.00603 (16)	−0.00168 (14)
C1	0.0158 (16)	0.0182 (13)	0.0147 (15)	−0.0033 (11)	−0.0014 (12)	−0.0026 (11)
C2	0.0111 (13)	0.0139 (11)	0.0148 (17)	−0.0035 (9)	−0.0047 (11)	0.0012 (10)
C3	0.0191 (16)	0.0228 (14)	0.0154 (15)	−0.0084 (12)	−0.0061 (13)	0.0028 (11)
C4	0.0186 (16)	0.0148 (13)	0.0246 (17)	−0.0040 (11)	−0.0077 (14)	0.0051 (12)
C5	0.0162 (15)	0.0147 (11)	0.0258 (14)	0.0011 (10)	−0.0031 (16)	−0.0051 (12)
C6	0.0130 (16)	0.0177 (12)	0.0151 (14)	−0.0012 (10)	−0.0021 (11)	−0.0001 (11)
C7	0.0099 (14)	0.0172 (12)	0.0118 (14)	−0.0022 (10)	−0.0003 (11)	0.0005 (11)
C8	0.0117 (15)	0.0122 (12)	0.0161 (15)	0.0003 (10)	−0.0025 (12)	0.0009 (11)
C9	0.0127 (14)	0.0186 (12)	0.0128 (13)	0.0002 (10)	−0.0016 (14)	−0.0050 (12)
C10	0.0140 (15)	0.0229 (13)	0.0071 (13)	−0.0014 (11)	−0.0001 (11)	0.0019 (11)
C11	0.0119 (14)	0.0159 (12)	0.0176 (18)	0.0004 (10)	−0.0017 (11)	0.0051 (10)
C12	0.0106 (14)	0.0176 (12)	0.0145 (13)	0.0000 (11)	−0.0009 (12)	0.0013 (10)
C13	0.0091 (14)	0.0147 (12)	0.0123 (14)	0.0014 (10)	−0.0006 (12)	0.0004 (10)
C14	0.0213 (16)	0.0132 (11)	0.0166 (14)	0.0005 (10)	0.0031 (13)	−0.0004 (11)
C15	0.0249 (17)	0.0213 (13)	0.0357 (18)	−0.0018 (11)	−0.0050 (16)	−0.0010 (14)
C16	0.0267 (16)	0.0190 (13)	0.0162 (16)	−0.0006 (11)	−0.0005 (14)	−0.0027 (11)
C17	0.0183 (15)	0.0101 (11)	0.0159 (13)	−0.0032 (11)	0.0042 (13)	0.0015 (10)
C18	0.0292 (18)	0.0126 (12)	0.0219 (16)	0.0034 (11)	−0.0003 (14)	−0.0041 (11)
C19	0.0277 (16)	0.0200 (12)	0.0182 (14)	0.0008 (11)	−0.0032 (15)	−0.0047 (13)
C20	0.0186 (15)	0.0148 (12)	0.0224 (15)	0.0015 (12)	0.0000 (15)	0.0052 (11)
C21	0.0271 (17)	0.0166 (13)	0.0206 (17)	0.0048 (11)	0.0045 (13)	−0.0051 (11)
N1	0.0144 (13)	0.0154 (11)	0.0149 (13)	−0.0001 (9)	0.0004 (10)	0.0007 (9)
N2	0.0160 (12)	0.0145 (10)	0.0146 (12)	−0.0005 (8)	0.0010 (12)	0.0021 (10)
N3	0.0157 (13)	0.0151 (10)	0.0198 (12)	0.0015 (9)	0.0020 (12)	0.0012 (9)
O1	0.0279 (12)	0.0150 (8)	0.0172 (10)	0.0034 (7)	0.0064 (10)	0.0036 (8)

### Geometric parameters (Å, º)

**Table:**

Br1—C20	1.905 (3)	C11—C13	1.419 (4)
C1—C3	1.384 (3)	C12—N3	1.285 (3)
C1—C2	1.396 (3)	C12—C13	1.459 (3)
C1—H1	0.95	C12—H12	0.95
C2—C6	1.395 (3)	C14—N3	1.470 (3)
C2—N2	1.435 (3)	C14—C15	1.524 (3)
C3—C4	1.376 (4)	C14—C17	1.524 (4)
C3—H3	0.95	C14—H14	1.0
C4—C5	1.397 (4)	C15—H15A	0.98
C4—H4	0.95	C15—H15B	0.98
C5—C6	1.386 (3)	C15—H15C	0.98
C5—H5	0.95	C16—C21	1.383 (4)
C6—H6	0.95	C16—C17	1.389 (3)
C7—C8	1.388 (3)	C16—H16	0.95
C7—C13	1.390 (3)	C17—C18	1.390 (4)
C7—H7	0.95	C18—C19	1.389 (4)
C8—C9	1.395 (3)	C18—H18	0.95
C8—N1	1.433 (3)	C19—C20	1.375 (3)
C9—C10	1.380 (3)	C19—H19	0.95
C9—H9	0.95	C20—C21	1.386 (4)
C10—C11	1.389 (3)	C21—H21	0.95
C10—H10	0.95	N1—N2	1.256 (3)
C11—O1	1.350 (3)	O1—H1A	0.84
			
C3—C1—C2	119.6 (2)	C7—C13—C12	120.1 (2)
C3—C1—H1	120.2	C11—C13—C12	121.1 (2)
C2—C1—H1	120.2	N3—C14—C15	108.0 (2)
C6—C2—C1	120.1 (2)	N3—C14—C17	109.8 (2)
C6—C2—N2	123.5 (2)	C15—C14—C17	112.67 (19)
C1—C2—N2	116.4 (2)	N3—C14—H14	108.8
C4—C3—C1	120.6 (3)	C15—C14—H14	108.8
C4—C3—H3	119.7	C17—C14—H14	108.8
C1—C3—H3	119.7	C14—C15—H15A	109.5
C3—C4—C5	120.1 (2)	C14—C15—H15B	109.5
C3—C4—H4	120.0	H15A—C15—H15B	109.5
C5—C4—H4	120.0	C14—C15—H15C	109.5
C6—C5—C4	120.0 (3)	H15A—C15—H15C	109.5
C6—C5—H5	120.0	H15B—C15—H15C	109.5
C4—C5—H5	120.0	C21—C16—C17	122.5 (3)
C5—C6—C2	119.6 (2)	C21—C16—H16	118.7
C5—C6—H6	120.2	C17—C16—H16	118.7
C2—C6—H6	120.2	C16—C17—C18	117.4 (2)
C8—C7—C13	121.1 (2)	C16—C17—C14	119.9 (2)
C8—C7—H7	119.4	C18—C17—C14	122.6 (2)
C13—C7—H7	119.4	C19—C18—C17	121.3 (2)
C7—C8—C9	119.4 (2)	C19—C18—H18	119.3
C7—C8—N1	124.7 (2)	C17—C18—H18	119.3
C9—C8—N1	115.9 (2)	C20—C19—C18	119.3 (3)
C10—C9—C8	120.4 (2)	C20—C19—H19	120.3
C10—C9—H9	119.8	C18—C19—H19	120.3
C8—C9—H9	119.8	C19—C20—C21	121.2 (3)
C9—C10—C11	120.5 (2)	C19—C20—Br1	118.8 (2)
C9—C10—H10	119.8	C21—C20—Br1	120.02 (19)
C11—C10—H10	119.8	C16—C21—C20	118.2 (2)
O1—C11—C10	119.0 (2)	C16—C21—H21	120.9
O1—C11—C13	121.3 (2)	C20—C21—H21	120.9
C10—C11—C13	119.7 (2)	N2—N1—C8	114.3 (2)
N3—C12—C13	121.0 (2)	N1—N2—C2	113.1 (2)
N3—C12—H12	119.5	C12—N3—C14	118.3 (2)
C13—C12—H12	119.5	C11—O1—H1A	109.5
C7—C13—C11	118.8 (2)		
			
C3—C1—C2—C6	0.8 (4)	C21—C16—C17—C18	0.3 (4)
C3—C1—C2—N2	178.1 (2)	C21—C16—C17—C14	178.9 (2)
C2—C1—C3—C4	−1.0 (4)	N3—C14—C17—C16	176.7 (2)
C1—C3—C4—C5	1.2 (4)	C15—C14—C17—C16	56.2 (3)
C3—C4—C5—C6	−1.1 (4)	N3—C14—C17—C18	−4.8 (3)
C4—C5—C6—C2	0.9 (4)	C15—C14—C17—C18	−125.3 (3)
C1—C2—C6—C5	−0.8 (4)	C16—C17—C18—C19	−0.5 (4)
N2—C2—C6—C5	−177.9 (2)	C14—C17—C18—C19	−179.0 (2)
C13—C7—C8—C9	2.8 (4)	C17—C18—C19—C20	−0.4 (4)
C13—C7—C8—N1	−176.2 (2)	C18—C19—C20—C21	1.3 (4)
C7—C8—C9—C10	−3.3 (4)	C18—C19—C20—Br1	−177.8 (2)
N1—C8—C9—C10	175.8 (2)	C17—C16—C21—C20	0.6 (4)
C8—C9—C10—C11	1.4 (4)	C19—C20—C21—C16	−1.5 (4)
C9—C10—C11—O1	−179.2 (2)	Br1—C20—C21—C16	177.69 (19)
C9—C10—C11—C13	1.1 (4)	C7—C8—N1—N2	11.8 (4)
C8—C7—C13—C11	−0.3 (4)	C9—C8—N1—N2	−167.2 (2)
C8—C7—C13—C12	177.8 (2)	C8—N1—N2—C2	177.17 (19)
O1—C11—C13—C7	178.7 (2)	C6—C2—N2—N1	−16.5 (3)
C10—C11—C13—C7	−1.6 (4)	C1—C2—N2—N1	166.3 (2)
O1—C11—C13—C12	0.5 (4)	C13—C12—N3—C14	178.6 (2)
C10—C11—C13—C12	−179.8 (2)	C15—C14—N3—C12	−113.7 (3)
N3—C12—C13—C7	−177.4 (2)	C17—C14—N3—C12	123.1 (3)
N3—C12—C13—C11	0.7 (4)		

### Hydrogen-bond geometry (Å, º)

**Table:**

*D*—H···*A*	*D*—H	H···*A*	*D*···*A*	*D*—H···*A*
O1—H1*A*···N3	0.84	1.84	2.585 (3)	148
C12—H12···*Cg*1^i^	0.95	2.80	3.399 (3)	122
C10—H10···*Cg*1^ii^	0.95	2.74	3.415 (3)	128
C6—H6···*Cg*2^iii^	0.95	2.75	3.423 (3)	128

Symmetry codes: (i) −*x*+1, −*y*+1, *z*; (ii) −*x*, −*y*+1, *z*+1; (iii) *x*, *y*, *z*+1.

## References

[bb1] Akitsu, T. (2007). *Polyhedron*, **26**, 2527–2535.

[bb2] Akitsu, T. & Einaga, Y. (2005*a*). *Polyhedron*, **24**, 1869–1877.

[bb3] Akitsu, T. & Einaga, Y. (2005*b*). *Polyhedron*, **24**, 2933–2943.

[bb4] Akitsu, T. & Einaga, Y. (2006*a*). *Polyhedron*, **25**, 1089–1095

[bb5] Akitsu, T. & Einaga, Y. (2006*b*). *Acta Cryst.* E**62**, o4315–o4317.

[bb6] Akitsu, T. & Itoh, T. (2010). *Polyhedron*, **29**, 477–487.

[bb7] Akitsu, T., Takeuchi, Y. & Einaga, Y. (2004). *Acta Cryst.* C**60**, o801–o802. 10.1107/S0108270104017354 15528824

[bb8] Aritake, Y., Watanabe, Y. & Akitsu, T. (2010). *Acta Cryst.* E**66**, o749. 10.1107/S1600536810007762 PMC2983984 21580594

[bb9] Aslantaş, M., Kurtoğlu, N., Şahin, E. & Kurtoğlu, M. (2007). *Acta Cryst.* E**63**, o3637.

[bb10] Bruker (1998). *APEX2* and *SAINT*. Bruker AXS Inc., Madison, Wisconsin, USA.

[bb11] Hadjoudis, E. & Mavridis, I. M. (2004). *Chem. Soc. Rev.* **33**, 579–588. 10.1039/b303644h 15592623

[bb12] Khandar, A. A. & Rezvani, Z. (1999). *Polyhedron*, **18**, 129–133.

[bb13] Miura, Y., Aritake, Y. & Akitsu, T. (2009). *Acta Cryst.* E**65**, o2381. 10.1107/S1600536809035557 PMC2970391 21577845

[bb14] Parsons, S., Flack, H. D. & Wagner, T. (2013). *Acta Cryst.* B**69**, 249–259. 10.1107/S2052519213010014 PMC3661305 23719469

[bb15] Sheldrick, G. M. (1996). *SADABS*. University of Göttingen, Germany.

[bb16] Sheldrick, G. M. (2008). *Acta Cryst.* A**64**, 112–122. 10.1107/S0108767307043930 18156677

[bb17] Sheldrick, G. M. (2015). *Acta Cryst.* C**71**, 3–8.

