# Supplementary figures and images for: Crystal structure of 2-{(R)-[1-(4-bromo­phen­yl)eth­yl]imino­meth­yl}-4-(phenyl­diazen­yl)phenol, a chiral photochromic Schiff base
Source: Acta Crystallogr E Crystallogr Commun. 2015 Oct 28;71(Pt 11):o886–7. doi: 10.1107/S2056989015019866 (PMC4645026; doi:10.1107/S2056989015019866)

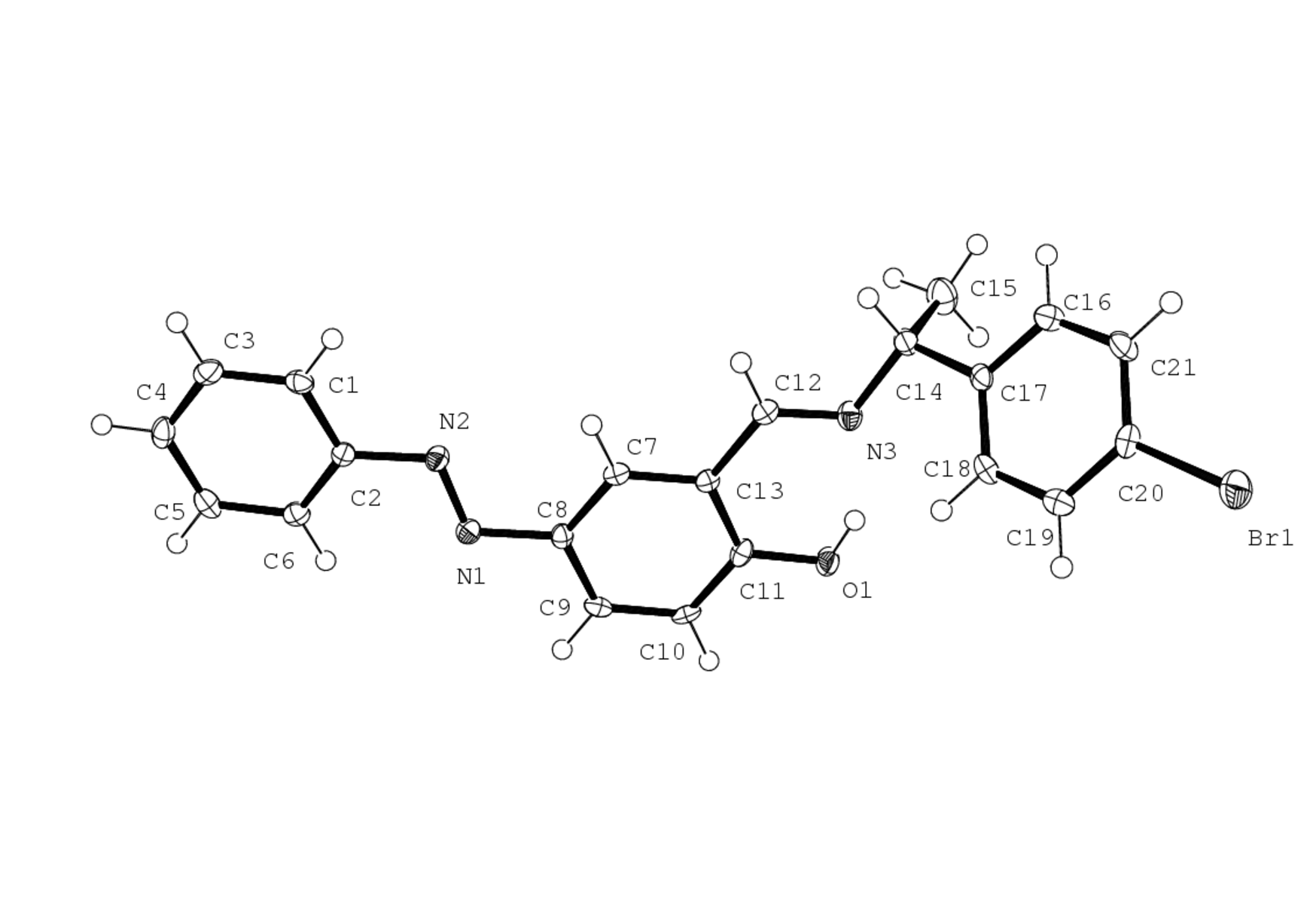

Supplement: Supplementary file 4 [file e-71-0o886-fig1.tif]
